# Output planning at the input stage in visual working memory

**DOI:** 10.1126/sciadv.abe8212

**Published:** 2021-03-24

**Authors:** Sage E. P. Boettcher, Daniela Gresch, Anna C. Nobre, Freek van Ede

**Affiliations:** 1 Oxford Centre for Human Brain Activity, Wellcome Centre for Integrative Neuroimaging, Department of Psychiatry, University of Oxford, Oxford, UK.; 2 Department of Experimental Psychology, University of Oxford, Oxford, UK.; 3 Department of Experimental Psychology, Ludwig-Maximilians-Universität München, Munich, Germany.; 4 Institute for Brain and Behavior Amsterdam, Department of Experimental and Applied Psychology, Vrije Universiteit Amsterdam, Netherlands.

## Abstract

Working memory serves as the buffer between past sensations and future behavior, making it vital to understand not only how we encode and retain sensory information in memory but also how we plan for its upcoming use. We ask when prospective action goals emerge alongside the encoding and retention of visual information in working memory. We show that prospective action plans do not emerge gradually during memory delays but are brought into memory early, in tandem with sensory encoding. This action encoding (i) precedes a second stage of action preparation that adapts to the time of expected memory utilization, (ii) occurs even ahead of an intervening motor task, and (iii) predicts visual memory–guided behavior several seconds later. By bringing prospective action plans into working memory at an early stage, the brain creates a dual (visual-motor) memory code that can make memories more effective and robust for serving ensuing behavior.

## Teaser

The human brain immediately plans for prospective action when storing sensations into working memory.

## INTRODUCTION

A fundamental purpose of the brain is to guide adaptive future behavior (*1*–*5*). Working memory plays a key role herein, as it enables representations of past sensations to inform and guide future behavior (*6*–*10*). To understand adaptive behavior, it is thus important to understand not only how we selectively encode and retain sensory information in working memory (*11*–*13*) but also how we plan for anticipated future memory use (*14*–*18*).

One fundamental purpose for holding detailed sensory information in working memory is to guide action (*15*, *19*–*25*). In accordance, recent work has shown that when memorized visual information is probed for guiding behavior, action plans can be selected from memory concurrently with probed visual representations (*26*). It remains an open question, however, when the plans for prospective actions are assembled before the moment memories become relevant for guiding behavior—i.e., alongside the encoding and retention of sensory information in working memory. Prospective action plans may emerge gradually after the encoding of sensory information [similar to the type of action preparation that has been observed preceding voluntary movement (*27*, *28*) or alongside perceptual evidence accumulation (*29*, *30*)]. Alternatively, prospective action plans may be brought into memory immediately, in tandem with sensory encoding (i.e., output planning at the input stage). To address this, we tracked neural planning for prospective manual actions during and following the selective encoding of visual orientation information into working memory.

Results from two experiments converged on a common insight: as the brain selects visual information to encode into working memory [input gating (*11*–*13*)], it already prospects the associated manual action that will become relevant at the end of the delay [output planning (*15*, *16*, *23*), cf. (*18*)]. We show that this action encoding into memory (i) is the first of two functional stages of planning where the brain first prospects what to do with the visual information that is being encoded in working memory, before gradually preparing for when to act on it (experiment 1); (ii) occurs even in the face of an intervening visual-motor task that discourages the preactivation of the action for upcoming execution (experiment 2); and (iii) is behaviorally relevant—predicting memory-guided behavior several seconds later (experiments 1 and 2).

## RESULTS

**Fig. 1:**
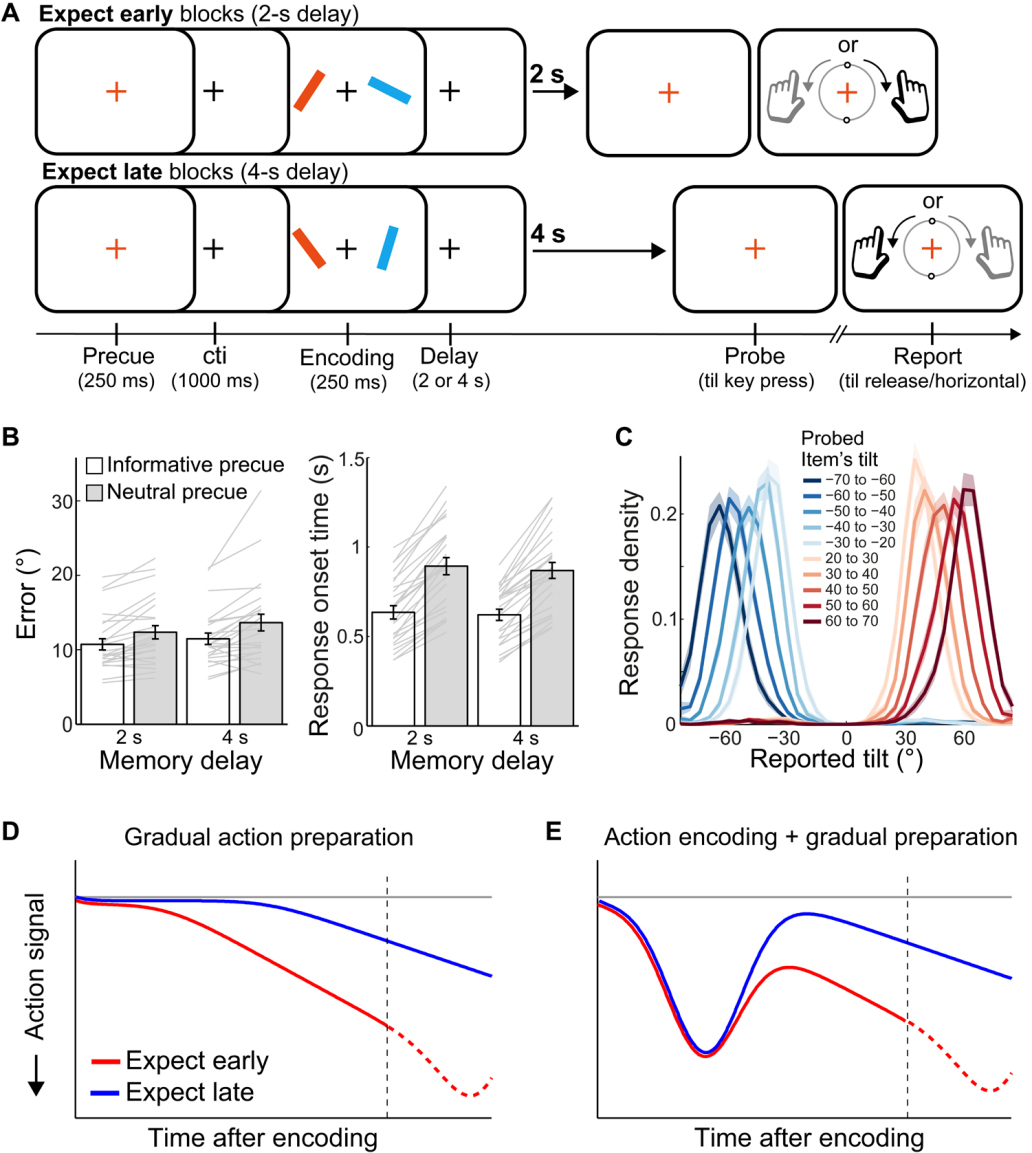
Task, behavioral performance, and hypothetical scenarios (experiment 1). (**A**) Task schematic of experiment 1. Trials started with a precue that could be either informative (80%; the color change of the fixation cross indicated with 100% validity which item would be probed) or neutral (20%; the cross turned gray, such that both memory items were equally likely to be probed). After a predictable memory delay of 2 or 4 s (blocked), participants reproduced the tilt of the memory item that was indicated by the color change of the central fixation cross. Item tilt was directly linked to the required action, such that a left/right-tilted item required reproduction with the left/right response hand (while required response duration depended on the detailed memory representation of tilt magnitude). Item location and required response hand were manipulated orthogonally. The example trials depict two left-item trials that required either a right (top) or left (bottom) hand response at the end of the memory delay. CTI, cue-target interval. (**B**) Reproduction errors and response times as a function of precue informativeness and memory delay. (**C**) Average response density as a function of the reported tilt and the tilt of the probed item. Zero degrees denote vertical and negative (positive) values denote a leftward (rightward) tilt. Error bars and shadings indicate ±1 SEM calculated across participants. Gray lines in (B) denote individual participants (*n* = 25). (**D** and **E**) Hypothetical patterns of prospective action preparation as a function of time of expected memory utilization.

Participants held visual shapes (tilted, colored bars) in working memory to reproduce their precise tilt after a multisecond delay. To promote the selective visual encoding of information into working memory, we presented informative precues. Before encoding, a brief color change of the central fixation cross would indicate which of the two ensuing visual items would be probed for reproduction after the memory period (Fig. 1A). To track prospective action preparation during this task, we linked visual tilts directly to response hands [as in (*26*)], such that a left/right-tilted bar (in memory) required tilt reproduction at the end of the memory delay with the left/right hand—while relying on precise visual orientation representation to reproduce the precise tilt (see Materials and Methods for details). Through the independent manipulation of the location and the tilt of the visual items, it was possible to track in parallel the temporal dynamics of encoding item location (a purely visual memory attribute in our task) and of its associated prospective manual action, using noninvasive human electroencephalography (EEG) measurements [as also in (*26*)].

In experiment 1, we varied the memory delay in a predictable manner (2 or 4 s in interleaved blocks; Fig. 1A), while in experiment 2, we inserted a secondary visual-motor task during the memory delay. As elaborated below, each of these manipulations helped disambiguate early encoding of the prospective action from the gradual development of action preparation (for ensuing execution) during the memory delay.

### Filtering relevant information into working memory improves future memory-guided action

We first confirmed that participants used the precues to selectively encode the relevant visual item into working memory. To this end, 80% of trials in experiment 1 contained an informative precue, while 20% of trials contained a neutral precue in which the fixation cross changed to gray. Using a 2 × 2 analysis of variance (ANOVA) with precue informativeness (informative versus neutral) and memory interval (2 s versus 4 s) as factors, we confirmed that informative precues facilitate later performance (Fig. 1B), as shown by lower reproduction errors [*F*(1,24) = 27.52, *P* < 0.001, η*_p_^2^* = 0.68] and faster response onsets after the probe [*F*(1,24) = 151.53, *P* < 0.001, η*_p_^2^* = 0.99]. Memory interval affected errors, with more errors after the longer delay [*F*(1,24) = 10.85, *P* = 0.003, η*_p_^2^* = 0.381] but had no effect on response times [*F*(1,24) = 1.58, *P* = 0.220, η*_p_^2^* = 0.304]. Precue informativeness and memory interval did not interact significantly for error [*F*(1,24) = 1.06, *P* = 0.314, η*_p_^2^* = 0.042] or response time [*F*(1,24) = 0.85, *P* = 0.365, η*_p_^2^* = 0.034]. Having confirmed the efficacy of the cue, all further analyses concern only those memory trials with an informative precue.

To confirm the use of the visual orientation information for guiding action, we looked at the profile of behavioral responses. As in our previous study (*26*), participants’ actions varied systematically with the memorized visual tilt, reproducing both the tilt direction and the tilt magnitude of the probed memory item (Fig. 1C).

These data thus confirm the utility of the precues and the use of the visual orientation information for guiding action. This provides a foundation for tracking visual selection and action preparation associated with the cued memory item in the EEG. We thus next turned to our main question regarding the dynamics of prospective action preparation during the memory period.

### Hypothetical scenarios of prospective action preparation alongside visual working memory

We considered two alternative scenarios by which prospective action plans are assembled during and/or following the selective encoding of visual information into working memory, as depicted in Fig. 1 (D and E). Because the neurophysiological markers of action preparation of interest are the attenuation (decrease) of 8- to 12-Hz mu-alpha and 13- to 30-Hz mu-beta activity (*31*–*34*), we depict action preparation negatively in our schematics.

In the first scenario, action preparation (alongside visual memory retention) emerges and develops gradually during the memory delay, becoming increasingly stronger as the time of expected memory-guided behavior approaches (Fig. 1D). This is similar to the type of action preparation that has been observed preceding voluntary movement (*27*, *28*) or alongside perceptual evidence accumulation (*29*, *30*). In this scenario, participants would select the relevant visual information to retain in working memory and thereafter gradually prepare the prospective action that is associated with this visual information. Alternatively, participants may bring the prospective action into working memory already at the visual encoding stage (action encoding), before gradually preparing to execute it (Fig. 1E). This second scenario uniquely predicts two stages of action planning and preparation, with an early action-encoding phase that occurs irrespective of the time of expected memory utilization (planning what to do at the end of the visual working memory delay), followed by a gradual preparation phase that is tuned to the time of expected memory utilization (preparing when to do it). This can thus be thought of as a what-then-when scenario of prospective action planning that may occur alongside the retention of visual orientation information in working memory. We provide multiple complementary sources of evidence for this action-encoding scenario, with two separate stages of planning.

### Prospective action preparation co-occurs with visual working memory

First, we tested whether action preparation occurred alongside retention of visual orientation information in working memory. To this end, we combined expect-early and expect-late trials and looked for neural signatures of selective visual and motor processing during two task periods: during the memory delay and following the memory probe.

We used amplitude modulations of posterior alpha activity as a signature of selective visual engagement (*35*–*37*) and central mu-alpha and mu-beta activity as a signature of motor engagement (*31*–*34*). We focused, respectively, on lateralized modulations that were sensitive to the selection of visual information at the item location and to the selection of the appropriate prospective response hand.

**Fig. 2:**
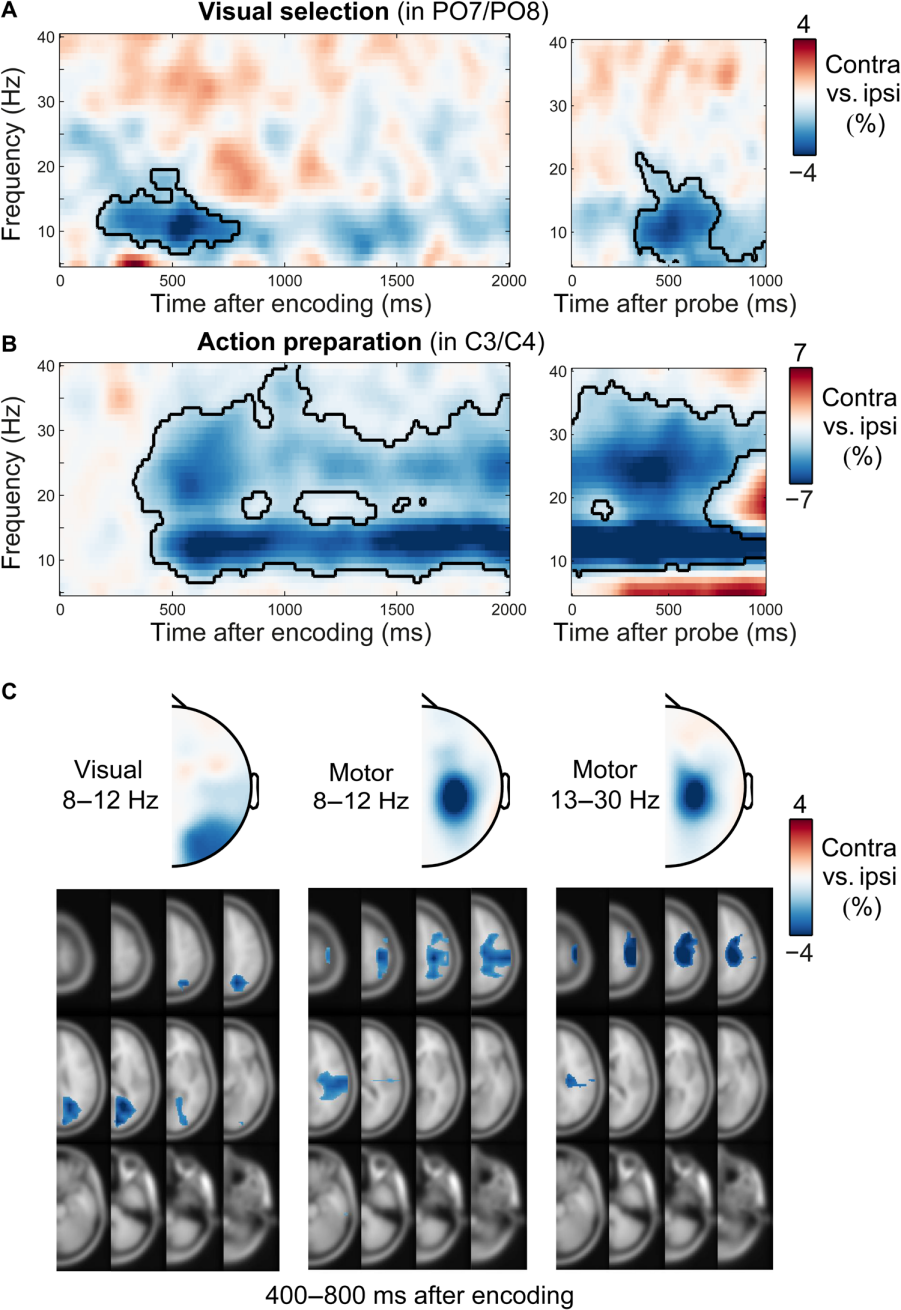
Prospective action preparation co-occurs with visual working memory. (**A**) Lateralized neural activity relative to the visual location of the cued (left) and probed (right) memory item. Visual lateralization was calculated in canonical visual electrodes (PO7/PO8). (**B**) Lateralized neural activity relative to the prospective response hand associated with the cued (left) and probed (right) memory item. Motor lateralization was calculated in canonical motor electrodes (C3/C4). Note how the visual location (visual) and prospective response hand (motor) were independently manipulated, yielding independent neural contrasts between (A) and (B). The black outlines indicate significant clusters following a cluster-based permutation analysis (*63*). (**C**) Sensor- and source-level lateralization relative to the visual location of the cued memory item (left, visual alpha) and to the prospective manual action associated with this item (middle, mu-alpha; right, mu-beta). Data from the 400- to 800-ms window after encoding onset (fig. S1 for a time-resolved topographical analysis that also includes the postprobe windows). Contralateral (contra) versus ipsilateral (ipsi) contrast values were projected into the right sensor/hemisphere of each symmetrical electrode/voxel pair, to match the corresponding time-frequency contrasts. Brain-slice depth runs from top left to bottom right in 12 steps. For visualization, a masking threshold was applied to all source values smaller than 50% of the maximum absolute contrast value.

In line with previous studies (*38*, *39*), lateralization of neural activity relative to the visual location of the selected memory item (Fig. 2A) showed a clear alpha attenuation in contralateral (versus ipsilateral) visual electrodes. This effect was particularly pronounced early during the memory delay (cluster *P* = 0.0036) and regained prominence after the probe (Fig. 2A, probe-locked data; cluster *P* = 0.0018). Because item location was a purely visual feature in our task, this shows that participants had retained visual information during the memory delay and used this information to guide their actions, as per our prior study (*26*).

Critically, our task enabled us to investigate not only the neural lateralization relative to the visual location of the relevant memory item but also relative to the prospective manual action that was associated with this item. This revealed a clear attenuation of spectral power in contralateral (versus ipsilateral) motor electrodes spanning the classical mu-alpha and mu-beta frequency bands [Fig. 2B; in line with (*19*, *31*–*33*)]. This effect emerged early in the memory delay (cluster *P* < 0.0001) and remained present throughout the delay, as well as after the probe (cluster *P* < 0.0001). Figure S1A shows an overlay of the time courses of these spatially selective visual and motor signatures.

Topographical analyses confirmed that the visual signatures localized to posterior (visual) EEG electrodes and cortical brain areas, while motor signatures localized to central (motor) electrodes and brain areas (Fig. 2C). Topographies remained stable throughout the course of the memory delay, as well as during the delay and after the probe (fig. S1B).

These data thus revealed that response hand–specific action preparation occurred prospectively alongside the retention of visual information in working memory. To disambiguate gradual action preparation from putative action encoding (see scenarios in Fig. 1, D and E), we next considered prospective action preparation as a function of the time of expected memory utilization.

### Action encoding into working memory followed by gradual action preparation

**Fig. 3:**
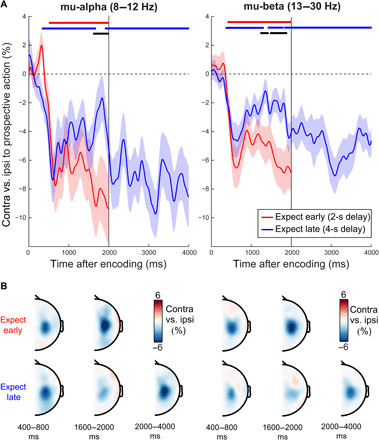
Early encoding followed by gradual preparation of prospective action. (**A**) Time courses of lateralized (effector-specific) action preparation signatures in mu-alpha (left) and mu-beta (right) separated according to the time of expected memory utilization and plotted up to the time of probe onset (2 and 4 s, respectively). Lateralization linked to action preparation was calculated in canonical motor electrodes (C3/C4). Horizontal lines indicate significant clusters, with the black lines denoting the difference between the expect-early and expect-late conditions (all clusters, *P* < 0.05). Shadings indicate ±1 SEM calculated across participants (*n* = 25). (**B**) Topographies at representative time windows in (A).

Figure 3 shows the mu-alpha and mu-beta neural signatures of prospective action preparation when the time of expected memory-guided behavior is 2 s (red) or 4 s (blue) after visual encoding (fig. S2 for the corresponding visual modulations). This revealed the characteristic biphasic profile of effector-specific action preparation described above. That is, there was an initial encoding of the prospective action followed by a period of gradual preparation. The pattern is particularly discernible in trials with a 4-s memory delay (blue time courses in Fig. 3A). While the initial mu-alpha and mu-beta modulations occurred largely independently of the time of expected memory utilization (each peaking within 400 to 800 ms after encoding-display onset), these signatures subsequently adapted to the time of expected memory utilization (black horizontal lines in Fig. 3A indicate significant differences between expect-early and expect-late time courses; mu-alpha, cluster *P* = 0.0014; mu-beta clusters, *P* = 0.048 and 0.0034). The pattern matches the schematic in Fig. 1E, whereby the brain encodes what action the selected visual information will serve (regardless of expected probe time) and subsequently prepares the action according to when it will become required. The early encoding and the later gradual preparation effects had similar central motor topographies (Fig. 3B).

### Action encoding occurs despite an intervening motor task

**Fig. 4:**
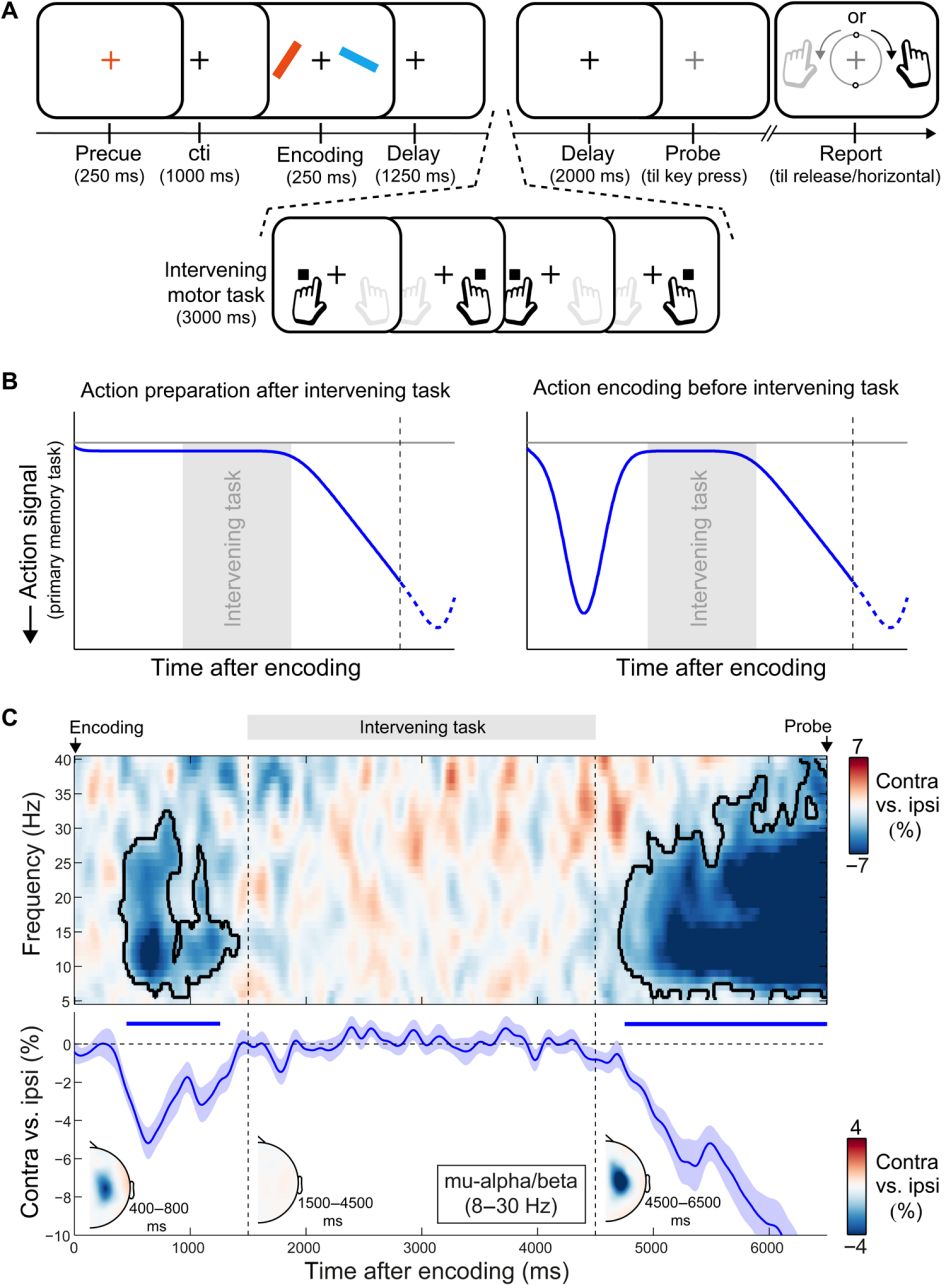
Action encoding occurs despite an intervening visual-motor task (experiment 2). (**A**) Task schematic of experiment 2. Participants performed a dual task with a primary memory task (like in experiment 1) and an intervening visual-motor task in the memory delay. The intervening task occurred on each trial and always consisted of two left and two right sequentially presented visual squares that indicated participants should press the corresponding button on the keyboard (using the same fingers and buttons that were used for the primary memory task). The first visual stimulus of the intervening task always occurred 1500 ms after encoding onset, while the last visual stimulus always occurred 4500 ms after encoding onset [as marked in (C)]. (**B**) Hypothetical patterns of action preparation in this task. (**C**) Neural lateralization in motor electrodes C3/C4 relative to the prospective response hand associated with the cued memory item in the primary memory task, across all frequencies (top), as well as for the 8- to 30-Hz band that spanned both the mu-alpha and the mu-beta bands (bottom). Insets show topographies at representative time windows. The black outlines and blue horizontal lines indicate significant clusters (all clusters, *P* < 0.05).

If action encoding reflects the process of encoding the prospective action into memory (rather than preactivating the action for upcoming execution per se), then action encoding should occur even when the action cannot yet be prepared for execution. To address this, in experiment 2, we inserted an intervening visual-motor task in the delay of each trial (Fig. 4A). This secondary task required participants to respond to four sequentially presented visual squares (two left and two right; in random order) with the corresponding hand. Accordingly, in this experiment, preparation for action execution in the primary memory task would not be advantageous until after the intervening task. We ask whether the first of the two identified stages of action planning—the action-encoding stage—would still come into play in this scenario.

We again considered two potential scenarios. First, action preparation for the primary visual working memory task might be upheld by the prospect of the intervening motor task. In this case, action preparation should commence only after the intervening task is completed; in line with a preparing-for-action-execution account (Fig. 4B, left scenario). Alternatively, prospective action plans may be brought into working memory during visual encoding despite the intervening task (Fig. 4B, right scenario) and be reinstated after the intervening task in anticipation of the memory probe that will prompt the visual working memory–guided action.

Figure 4C provides clear evidence for this second scenario. We found robust encoding of the prospective action for the primary memory task before the intervening task [cluster *P* (time-frequency map, top) = 0.008; (time course, bottom) = 0.004], followed by a pronounced reinstatement of action preparation soon after the final beat of the intervening task [cluster *P* (time-frequency map, top) < 0.0001; (time course, bottom) < 0.0001]. This was again similar for the mu-alpha and mu-beta bands, so we have averaged their time courses for visualization (Fig. 4C). As in experiment 1, the early encoding and later gradual preparation effects each had lateralized central topographies (Fig. 4C, insets) that localized to motor cortex (fig. S3).

Note that the absence of any prospective action preparation during the intervening task itself does not imply that the motor system was not active during this time period. Quite the contrary, participants correctly responded to 98.35 ± 0.32 (mean ± SEM) % of the visual squares, with an average reaction time (RT) of 311.67 ± 4.19 ms. Moreover, we found profound neural signatures of action, when considering neural lateralization relative to the manual responses in this secondary task (fig. S4). Instead, Fig. 4C shows that our primary action preparation signature (that is specific to the prospective response hand that will become relevant for the primary memory task) does not persist during the intervening task but rather is initially encoded into memory and brought back from memory upon completing the intervening task.

### Action encoding reflects anticipated future task demands, not the visual stimulus

**Fig. 5:**
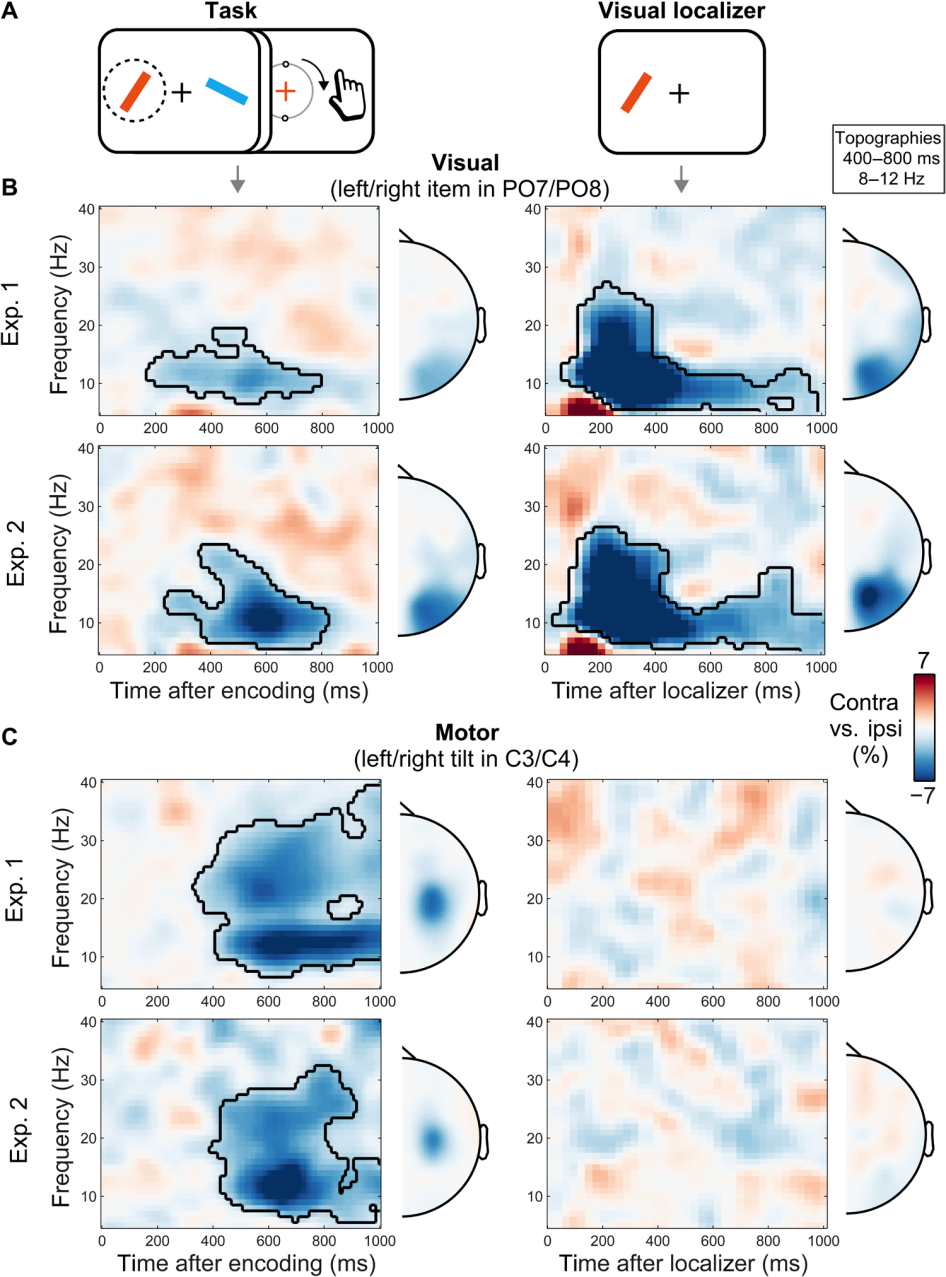
Action encoding reflects the anticipated task, not the visual stimulus. (**A**) Schematic of the working memory task and the task-free visual localizer. The circled item in the task schematic indicates the cued memory item. (**B**) Neural lateralization in visual electrodes relative to the location of the visual item in the task (left) and in the task-free localizer (right). Data from experiments 1 (top) and 2 (bottom). (**C**) Neural lateralization in motor electrodes relative to the tilt of the visual item in the task (left) and in the task-free localizer (right) in experiment 1 (top) and 2 (bottom). In the task, visual tilt was linked to the prospective response hand, whereas this was not the case in the task-free localizer. Visual localizer modules occurred in between task blocks and contained sequentially presented left and right bars with left and right tilts; identical to the items used in the task, except presented in isolation. The black outlines indicate significant clusters (all clusters, *P* < 0.05). Conventions as per Figs. 2 and 4.

In both experiments, prospective manual actions were linked to the tilt of the selected visual items. To demonstrate that the observed action signatures reflect anticipated task demands (rather than visual tilt per se), we calculated equivalent contrasts following visual stimuli that were presented in isolation during visual localizer modules (inserted between task blocks) in which the participants’ only task was to maintain fixation (Fig. 5A). We observed strong modulations by the location of the visual stimuli in visual electrodes in the memory tasks in experiments 1 and 2 (Fig. 5B, left) as well as in the task-free localizers (Fig. 5B, right). Critically, however, in contrast to the pattern of action encoding associated with the visual tilt that we observed during the two memory tasks (Fig. 5C, left), there was no equivalent action signature induced by the tilt of the visual stimuli in motor electrodes during the localizers (Fig. 5C, right). This pattern of results was highly replicable between experiments. Thus, the observed action encoding is not a passive consequence of the visual attributes of the memory material but rather reflects context-dependent action planning in service of anticipated future demands that are specific to the memory task.

### Action encoding predicts later memory-guided behavior

**Fig. 6:**
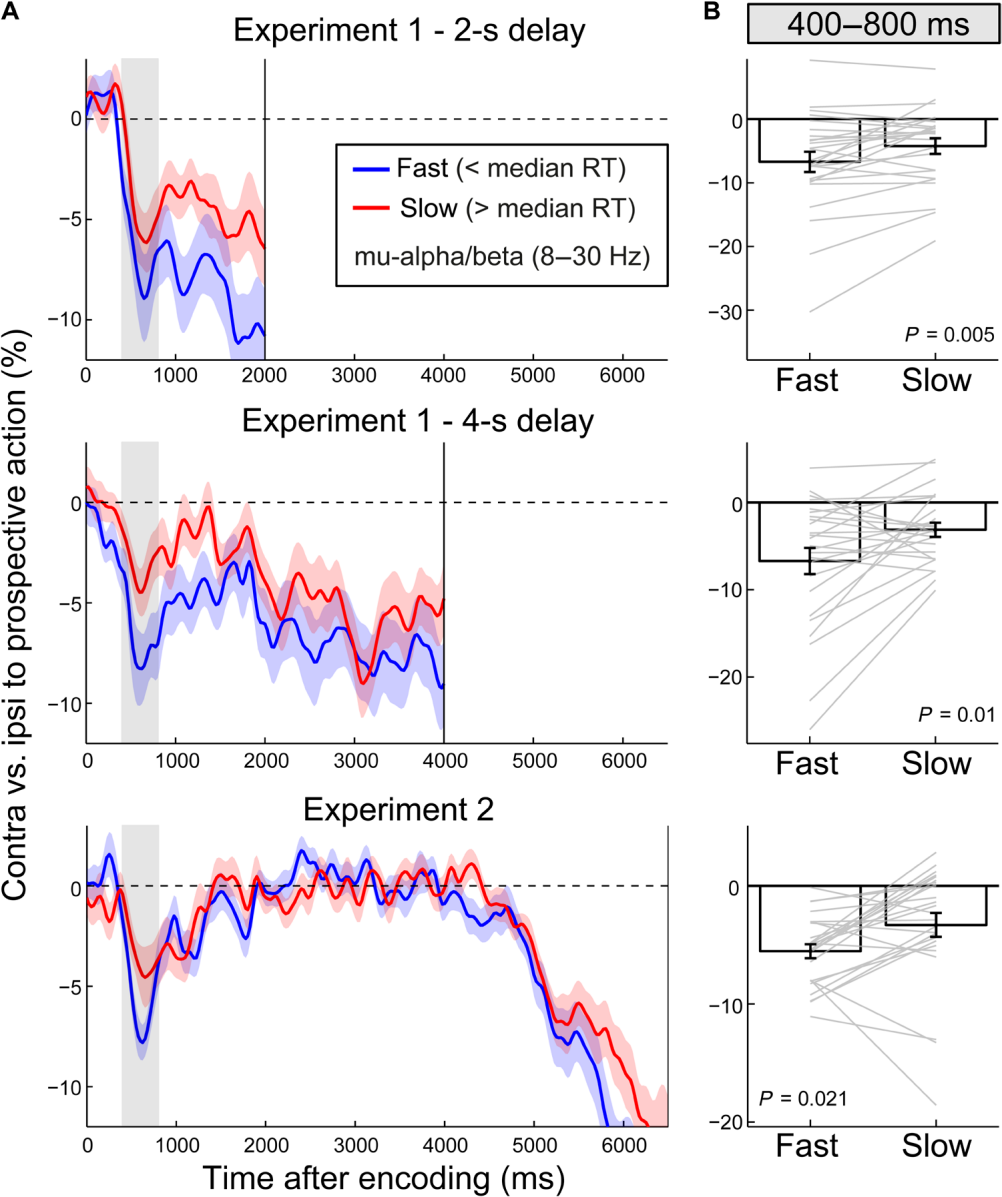
Action encoding predicts response times of memory-guided behavior after a multisecond delay. (**A**) Prospective action preparation (8- to 30-Hz lateralization in C3/C4 relative to the prospective manual action) as a function of response times after the memory delay (median split), separately for experiment 1 for blocks with a 2-s delay (top), a 4-s delay (middle), and experiment 2 (6.5-s delay, bottom). Mean response times for fast and slow trials were as follows: experiment 1 (2-s delay), 430 ± 19 (fast) and 848 ± 59 ms (slow) (means ± SEM); experiment 1 (4-s delay), 440 ± 18 and 813 ± 48 ms; experiment 2, 337 ± 17 and 668 ± 44 ms. (**B**) Action encoding for fast and slow trials, averaged across the 400- to 800-ms action-encoding window. This window was chosen a priori (based on the preceding results) and is also indicated in the gray shadings in (A). Error bars and shadings indicate ±1 SEM calculated across participants. Gray lines in (B) denote individual participants (*n* = 25).

As a last step, we tested whether the identified early action encoding had consequences for later memory-guided behavior (after a delay of 2 or 4 s in experiment 1 and 6.5 s in experiment 2). To this end, we first split the trials according to response times after the memory probe, defined as the time from probe onset to response initiation. Figure 6A shows prospective action preparation during the delay, when the delay period was followed by fast (< median) and slow (> median) responses. Notably, in each case, trials with faster responses were associated with stronger action encoding at the time of visual encoding, even when this encoding occurred more than 4 or even 6.5 s before the behavioral response and even when participants had completed a secondary task in the interim (experiment 2). When considering the 400- to 800-ms window in which action encoding was observed (Figs. 3A and 4C; also indicated in the shaded areas in Fig. 6A), we found significantly more pronounced action encoding in fast versus slow trials in each case [Fig. 6B; experiment 1 (2-s delay): *t*_(24)_ = −3.064; *P* = 0.005; *d* = −0.613; experiment 1 (4-s delay): *t*_(24)_ = −2.815; *P* = 0.01; *d* = −0.563; experiment 2: *t*_(24)_ = −2.479; *P* = 0.021; *d* = −0.496].

**Fig. 7:**
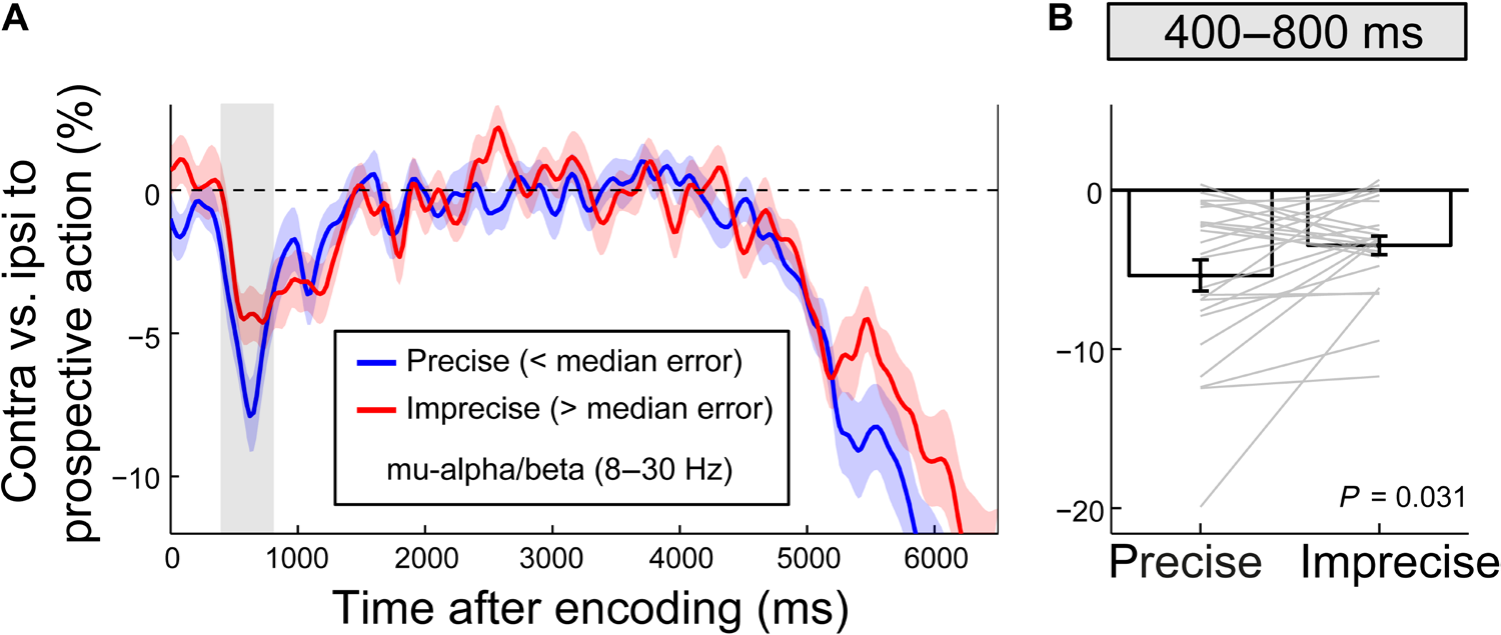
Action encoding predicts the precision of memory-guided behavior when working memory is interrupted by an intervening task. (**A**) Prospective action preparation (8- to 30-Hz lateralization in C3/C4 relative to the prospective manual action) as a function of the precision of the reproduction report in the working memory task at the end of the delay (median split) in experiment 2. Mean reproduction errors for precise and imprecise trials were 12.4° ± 1.6° and 29.6° ± 1.8°. (**B**) Action encoding for precise and imprecise trials, averaged across the 400- to 800-ms action-encoding window. This window was chosen a priori (based on the preceding results) and is also indicated in the gray shadings in (A). Error bars and shadings indicate ±1 SEM calculated across participants. Gray lines in (B) denote individual participants (*n* = 25).

By incorporating plans for prospective actions alongside visual representations at an early stage, the working memory trace may become more robust to subsequent interference. In support of this, when the working memory task was interrupted by the intervening task in experiment 2, we found that the early action-encoding effect (that occurred ahead of the intervening task) also predicted the quality of working memory–guided behavior (that occurred after the intervening task) [Fig. 7; *t*_(24)_ = −2.287; P = 0.031; *d* = −0.457]. While a similar link between early action encoding and the quality of memory-guided behavior could not be established in experiment 1 [2-s delay: *t*_(24)_ = 1.093; *P* = 0.285; *d* = 0.219; 4-s delay: *t*_(24)_ = −1.549; *P* = 0.134; *d* = −0.31], in experiment 1, there was no clear source of interference during the memory delay and there was overall less variability in the quality of memory-guided behavior (fig. S5).

Last, to assess whether the observed relationships between action encoding and subsequent performance are task specific, we also sorted the trials in experiment 2 by response times and accuracy on the intervening task rather than on the primary memory task as we had done in Figs. 6 and 7. This showed no clear differences in action encoding (for the primary memory task) in trials in which participants were fast versus slow [*t*_(24)_ = −0.864; *P* = 0.396; *d* = −0.173] or accurate versus inaccurate [*t*_(24)_ = −0.5603; *P* = 0.581; *d* = −0.112] on the intervening task (fig. S6). This suggests that the relations between action encoding and ensuing performance (Figs. 6 and 7) are task specific and are therefore unlikely driven by more general difference between trials such as differences in motivation or arousal.

## DISCUSSION

Because memories serve future behavior (*16*), processes by which we selectively encode relevant sensations into memory [input gating (*11*–*13*)] must be complemented by processes by which we prepare memoranda for their future use [output planning (*14*–*16*, *23*); cf. (*18*)]. Through careful experimental design, we tracked the neural signatures of both processes independently, revealing the insight that output planning starts at the input stage. Specifically, alongside selectively encoding visual information into working memory, the human brain prospects the manual action that this information will serve—as if imprinting the prospective action plan into memory for later use. Across two experiments, we have shown how this action encoding (i) is the first of two functional stages of planning where the brain first prospects what action will become required (given the encoded visual information), before preparing for when to execute it (experiment 1); (ii) occurs despite an intervening motor task that discourages the preactivation of the action for upcoming execution (experiment 2); and (iii) predicts memory-guided behavior several seconds later (experiments 1 and 2) and even when performing a secondary task in the interim.

Action encoding may serve at least two complementary purposes: making memories more effective and making them more robust. First, it ensures that our visual memories are ready for action such that, whenever memorized visual information becomes relevant for guiding behavior, the associated action is already available in memory (note that our task did not strictly require participants to encode and memorize the prospective action; participants could have relied on a purely visual memory trace and considered the action only after the probe). This action readiness would, in turn, yield faster memory-guided behavior; a prediction that we confirmed in our data, by showing that action encoding predicted faster response times after the memory delay. However, the utility of such action-embedded memories may go beyond a pure readiness account. Action encoding was independent of the expected probe time and occurred even when an intervening task negated the utility of preparing the action for ensuing execution. A second purpose of such action encoding may be to make memories more robust. By creating a dual memory code upon encoding—containing future action plans alongside detailed sensory information rather than either in isolation (*17*, *40*, *41*)—memories may become more resilient to subsequent interference [similar to the benefits observed by distributing memory traces across hemispheres (*42*, *43*)]. In line with this proposal, when there was a clear source of interference—in experiment 2 when the memory task would be interrupted by the intervening motor task—we found that the strength of the initial action-encoding effect predicted even the quality of later memory-guided behavior.

Our results build on and extend several lines of prior research that have linked visual working memory and action (*23*–*25*). For example, it has been established that overt actions can themselves influence visual working memory performance (*24*, *44*, *45*) and that brain structures that control action also participate in visual working memory (*46*, *47*). We investigated the complementary direction of this interaction, revealing that the selection of visual information into working memory also naturally recruits the preparation for prospective manual actions [see also (*19*–*22*)]. Moreover, by adopting a task in which actions required the guidance of detailed visual orientation information from memory, we also complement prior work that has focused on actions that required guidance from purely spatial locations from memory (*6*, *48*–*50*) or guidance from detailed visual information from perception (*51*–*53*).

Our results further demonstrate the flexible, context-dependent nature by which vision can inform action (*54*). Action encoding occurred in response to the visual memory items that were linked to future actions but not in response to identical visual items that required no action as part of a task-free localizer. This complements, and is distinct from, prior studies on the automatic activation of compatible motor codes (*5*, *55*) or on the role of action affordances (*1*, *56*) in visual working memory that focused on affordances inherent to the memory material itself [such as when retaining images of manipulable versus nonmanipulable objects (*57*)]. We show that the type of visually derived action associations that we studied here can become effective in a task-specific manner, being instantiated only when they are relevant for prospective task goals.

In the current work, we studied action planning alongside the retention of sensory representations in working memory. Related work from the perceptual decision-making literature has revealed how motor decisions also form during the accumulation of sensory evidence, ahead of the decision report (*29*, *30*, *58*, *59*). Our results complement this related line of research. For example, unlike in typical perceptual decision-making tasks in which noisy sensory evidence is gradually accumulated until a decision is reported, in our working memory task, the sensory evidence was clear immediately but had to be retained in memory for guiding behavior several seconds later. In this setting, we found immediate encoding of the prospective action, despite the fact that there was no strict need to encode the prospective action in this early period (because we used multisecond memory delays that were fully predictable, participants could have equally well gradually considered the relevant manual action over the course of the memory delay). In addition, behavioral responses in our working memory task pertained to precise orientation reproduction reports from memory, rather than a binary report of the decision, as is typical in perceptual decision-making tasks. Last, we found that the early action-encoding effect occurred even in face of the intervening task that would also tax the motor system. Whether early motor-decision signals would also occur in face of such intervening tasks in more traditional perceptual decision-making tasks remains an interesting question.

On the basis of the observed temporal profiles of action planning, we have proposed two stages of action planning during working memory. This interpretation of two functional stages is based primarily on experiment 1, whereby initial action encoding occurred irrespective of the expected probe time, whereas the second (preparation for execution) stage adapted to the expected time of memory-guided behavior. Experiment 2, in turn, confirmed the occurrence of this initial stage even when preparation for execution was discouraged by virtue of the intervening task. At the same time, in both experiments, these two temporal stages were associated with qualitatively similar neural signatures of attenuated mu-alpha and mu-beta activity. Thus, while the two stages could be disentangled by means of their functional properties—whereby only the second stage adapted to the expected time of memory-guided behavior—the underlying neural processes and computations may or may not be similar during both stages. On the basis of our scalp EEG signatures alone, it remains difficult to disentangle whether the observed bimodal pattern of action planning reflects a single action preparation process with a time-varying gain or instead reflects two qualitatively distinct states of planning that are also instantiated at different times.

By focusing on prospective actions, we have studied the mechanisms by which we prepare our memories to serve future behavior. This has painted a rich picture of the prospective and dynamic nature of working memory, whereby the prospective action goal is brought into memory before being titrated to the expected time of memory use. Other dimensions of prospective memory preparation involve, for example, the future location at which memories are expected to become relevant or the exact task demands that these memories are expected to serve (e.g., visual identification versus action guidance). Our insights pave the way for future studies to investigate the temporal profiles and neurophysiological basis of these complementary dimensions of memory preparation.

## MATERIALS AND METHODS

### Participants

The study was approved by the Central University Research Ethics Committee of the University of Oxford. Sample size for both experiments was set to *N* = 25 before data collection, to match our prior study which addressed a complementary question using a similar approach (*26*). In experiment 1, we collected data from 26 human volunteers ranging in age between 18 and 35 (mean age, 26.54 years; 19 females; all right-handed). One participant was discarded because she performed the task using transient key presses (rather than key presses of the required duration), making it impossible to tell whether she guided her actions by detailed visual representations or merely remembered which key to press. In experiment 2, we collected data from a new sample of 25 human volunteers ranging in age between 18 and 32 (mean age, 23.71 years; 17 females; one left-handed) and retained all datasets for analysis. All participants reported normal or corrected-to-normal vision, provided written consent before participation, and were reimbursed £15/hour.

### Experiment 1: Stimuli, task, and procedure

We used a recently developed visual-motor working memory task (*26*) that we here adapted to study action planning relative to the selective encoding and retention of information in working memory and that is shown in Fig. 1A (Fig. 4A for experiment 2). In short, participants had to report back (reproduce) the exact tilt of the probed (color-matching) memory item after a memory delay of either 2 or 4 s. Two defining aspects of this task are that (i) the item’s tilt is directly linked to the prospective action (response hand and response duration) required at the end of the memory delay and (ii) visual item location and the prospective response hand were manipulated orthogonally (and can therefore be tracked independently in the trial-average EEG signal). We return to these task features at the relevant instances below.

The experiment was programmed in Presentation (version 17.2, Neurobehavioral Systems Inc., Berkeley, CA). Visual-encoding displays consisted of two tilted bars of different color. One bar was always positioned on the left and the other on the right of the central fixation cross, centred at 5.7° visual angle. Independently of the bars’ locations, one of the bars was tilted to the left and the other to the right. The bars were 0.8° in width and 5.7° in length. To avoid tilts too close to the vertical and horizontal meridians, tilt direction was randomly drawn between ±20° and ±70°.

Across trials, a leftward (rightward) tilted bar was equally likely to appear in the left or right position. Consequently, bar tilt was independent of the bar location. In each trial, the colors of the two bars were drawn randomly—without duplication—from a set of four highly distinguishable colors: blue (RGB: 21, 165, 234), orange (RGB: 234, 74, 21), green (RGB: 133, 194, 18), and purple (RGB: 197, 21, 234).

Each trial started with the presentation of a precue in the form of a change in the color of the central fixation cross. This precue was 100% informative in the vast majority (80%) of trials and was neutral (uninformative) in the remaining trials. In informative precue trials, the central fixation cross briefly took the color of one of the two upcoming bars and predicted with 100% reliability that this item would be probed after the memory delay—thus enabling selection of the relevant item at visual encoding. The location of the precued item was not known until the visual encoding display appeared. In the neutral precue trials, the color of the fixation cross turned gray instead (RGB: 150, 150, 150), and the two bars were equally likely to be probed after the memory delay. Precues were displayed for 250 ms. The encoding display with the two bars appeared 1000 ms after precue onset and was presented for 250 ms. Visual encoding was followed by a memory delay, in which only the fixation cross remained on the screen. In different blocks, the memory delay was either 2000 or 4000 ms from the onset of the encoding display. After this memory delay, the fixation cross again changed color to indicate which of the items should be reported.

Prompted by the fixation cross color change at the end of the delay (referred to as the probe), participants reproduced the tilt of the color-matching bar as accurately as possible. For reproduction, participants were asked to press the “\” (backslash) or the “/” (forward slash) key on the keyboard using their left or right index finger, respectively. The time between probe onset and response initiation was unlimited. After response initiation, a dial appeared around fixation with the same diameter as the length of the bars. The response dial always began in the vertical position and moved at a speed of 1/8° per millisecond either clockwise (right response) or counter clockwise (left response). Participants were instructed to release their key press when the dial reached the tilt of the item in memory. Only one key could be used to reproduce the item’s tilt, and participants were not able to adjust their response after key release. Critically, because the response dial always started in the top vertical position and because it could not be rotated beyond ±90° (horizontal), a leftward (rightward) tilted bar could only be accurately reported with a left (right) key press. As a consequence, the hand required for responding was directly linked to the tilt of the bar that was probed; while a detailed visual representation remained relevant for guiding the precision (duration) of this manual reproduction report. Critically, because bar tilt and bar location were orthogonal across trials (a left/right bar equally often required a left/right hand response), it was possible to characterize independently neural activity reflecting the probed item’s memorized location (a purely visual memory attribute in our task) and the prospective response hand that was associated with the probed item’s tilt.

Upon key release, feedback was presented for 250 ms above the central fixation cross in the form of a number, ranging from 0 to 100 (with 100 indicating perfect report and 0 indicating that the reported tilt was perpendicular to the required report). Intertrial intervals were randomly drawn between 500 and 800 ms. To perform the task, participants sat in a dimly lit booth in front of a monitor [22-inch (55.88 cm) Samsung SyncMaster 2233; resolution, 1680 × 1050 pixels; refresh rate, 100 Hz; screen width, 47 cm] at a viewing distance of approximately 95 cm.

Before the start of the experiment, participants performed several practice trials to become familiar with the task. Experiment 1 was divided into two consecutive sessions, each of approximately 50 min, with a 10- to 15-min break between them. In each session, participants completed 10 blocks of 40 trials, yielding 800 trials in total.

Blocks with 2- and 4-s memory delays were randomly intermixed (with each session containing five of each). Memory delay was indicated on the screen before each block commenced and was 100% reliable. The location and tilt (response hand) of the memory items were pseudo-randomized across trials, such that each of the key conditions—the cued item being the left item with a leftward tilt, the left item with a rightward tilt, the right item with a leftward tilt, or the right item with a rightward tilt—occurred equally often in each block of 40 trials.

### Experiment 2

In experiment 2, we added an intervening secondary task in the delay period (Fig. 4A). The primary memory task followed the same procedure described above for experiment 1, with a few exceptions. Informative precues occurred in 100% of the trials. It was no longer necessary, therefore, to use a color-matching probe at the end of the delay, so we used a gray probe instead. Last, the memory delay was no longer varied but was 6.5 s in every trial.

In the intervening visual-motor task, four white squares (0.4° width and height) were presented sequentially to the left and right of fixation (at 5.7° visual angle). Participants were instructed to respond to these squares by pressing the corresponding left and right response keys within 500 ms. If participants failed to respond in time, or pressed the wrong key, the square flashed red for 100 ms as feedback. Responses were made with the same keys as those required for the primary memory task. The first square of the intervening task always occurred 1500 ms after onset of the encoding display for the primary memory task, while the last square always occurred exactly 3 s later (4500 ms after memory encoding). Times between square onsets varied between 800, 1000, and 1200 ms (in randomized order).

Like experiment 1, experiment 2 was divided into two consecutive sessions, each of approximately 50 min. In each session, participants completed seven blocks of 40 trials, yielding 560 trials in total. Experiment 2 had slightly fewer trials than experiment 1, due to the longer memory delays used in experiment 2.

### Visual localizer module

In both experiments, visual localizer modules were inserted, either after each task block (experiment 2) or pair of blocks (experiment 1). During the visual localizer, observers were instructed to relax and maintain fixation at the central fixation cross while tilted bars sequentially appeared for 100 ms with a randomly drawn interstimulus interval between 400 and 500 ms. Localizer stimuli appeared individually but were otherwise identical to the bars used in the task (i.e., localizer stimuli were between ±20° and ±70° tilt, presented at 5.7° visual angle to the left or right of fixation, in any of the four colors). Each visual localizer module contained 30 left and 30 right stimuli in randomized order in experiment 1 and 15 left and 15 right stimuli in experiment 2—yielding 600 localizer stimuli in experiment 1 and 420 in experiment 2, per participant.

### Analysis of behavioral data

Behavioral data were analyzed in MATLAB (version 2012b, MathWorks). Reproduction errors were calculated as the average absolute deviation between the probed item’s tilt and the reported tilt. Response times were calculated as the time from probe onset to response initiation. We only considered trials in which response initiation times were within 4 SD of the mean (98 ± 0.1% of trials in experiment 1 and 97 ± 0.2% of trials in experiment 2). In experiment 1, response times and reproduction errors were submitted to repeated-measures ANOVAs with the factors precue informativeness (informative/neutral) and working memory delay (2 or 4 s). For visualization, we also calculated response densities as a function of the probed item’s tilt. Response densities were quantified in bins of 10° (sampled in steps of 5° from −90° to +90°), separately for memory items with tilts between (−70 to −60), (−60 to −50), and so on.

### EEG acquisition

We acquired EEG using SynAmps amplifiers and Neuroscan data acquisition software (Compumedics). Sixty-one electrodes were distributed across the scalp using the international 10-10 positioning system. Data were referenced to the left mastoid during acquisition, and we included a right mastoid measurement to derive an average mastoid reference offline. The ground was positioned on the left upper arm. We concurrently collected vertical and horizontal electrooculography (EOG), using a bipolar montage with electrodes to the side of each eye, and above and below the left eye. During acquisition, data were filtered between 0.1 and 200 Hz, digitized at 1000 Hz, and stored for offline analysis. Electrode impedance was aimed to be below 5 kiloohm and was at least below 10 kiloohm.

### Analysis of EEG data

#### Preprocessing

EEG data were also analyzed in MATLAB using the FieldTrip toolbox (*60*). Independent component analysis (ICA) was used to correct for ocular artifacts in the EEG. Relevant ICA components were identified by correlating the component time courses with the measured horizontal and vertical EOG. A surface Laplacian transform (*61*) was applied to increase spatial resolution and interpretability of the 8- to 12-Hz and 13- to 30-Hz spectral modulations under consideration. Trials with exceptionally high variance were discarded following visual inspection (using the function “ft_rejectvisual” with the summary method). Trial removal was performed without knowledge of the experimental conditions to which individual trials belonged. After trial removal, 704 ± 16 of the total of 800 trials (88 ± 2%) were retained for analysis in experiment 1 and 496 ± 10 of the total of 560 trials in experiment 2 (90 ± 1%), per participant. Analysis windows were centred on the encoding of the visual display and on probe onset. All EEG analyses focused on the trials with an informative precue, where neural lateralization was defined relative to the visual location and to the prospective response hand associated with the cued item (trials with a neutral precue served only to confirm the use of the precue in the behavioral data).

#### Electrode and frequency-band selection

To investigate the lateralization of neural activity relative to the visual location (visual) and prospective response hand (motor) of the cued memory item, we focused our analyses on canonical left and right visual and motor EEG electrodes (PO7 for left visual, PO8 for right visual, C3 for left motor, and C4 for right motor). For the analysis of band-specific time courses, we used the predefined frequency bands that are equally conventional: 8 to 12 Hz for visual alpha and for mu-alpha (motor alpha) and 13 to 30 Hz for mu-beta (motor beta).

#### Time-frequency analysis

Time-frequency maps were calculated using a short-time Fourier transform of Hanning-tapered data. Spectral power was assessed for frequencies between 5 and 40 Hz (in steps of 1 Hz), using a 300-ms sliding time window that was advanced in steps of 10 ms. Activity in predefined visual (PO7/PO8) and motor (C3/C4) electrodes was contrasted between trials in which either the item (visual) or the prospective response hand (motor) was contralateral versus ipsilateral to the electrode of interest. We expressed this as a normalized difference [i.e., ((contra-ipsi)/(contra + ipsi)) × 100] and averaged these contrasts across the left and right electrodes (separately for visual and motor contrasts in visual and motor electrodes). Topographical maps of lateralization were obtained by applying the same procedure to all symmetrical electrode pairs (and were plotted in the right electrode of each pair). To extract time courses of alpha and beta lateralization from these time-frequency contrasts, we averaged across the predefined alpha band (8 to 12 Hz) for the item-location lateralization in the visual electrodes, and we averaged across the predefined mu-alpha (8 to 12 Hz) and mu-beta (13 to 30 Hz) bands for the prospective response hand lateralization in the motor electrodes.

Independence between our visual and motor signatures of interest is achieved through the fact that the visual and motor contrasts are orthogonal in the trial average. That is, these contrasts are selectively sensitive to the visual location or the prospective response hand (by virtue of the orthogonal manipulation of item location and item tilt). This is also appreciated by the observation that the topographies of these contrasts look fundamentally different, even for the exact same frequency band (Fig. 2C). The use of distinct electrodes for both contrasts, served to zoom in on the sites at which the respective modulations were hypothesized to occur (PO7/PO8 for the visual contrast and C3/C4 for the motor contrast).

#### Relation with behavior

To investigate the relation between prospective action preparation and eventual memory-guided behavior, we split the trials by their response initiation times after the probe, as well as by the precision of the reproduction report. We performed median split analyses separately for each experimental condition (left/right items associated with left/right actions), effectively regressing out low-level differences in response times due to item side and/or response hand. We only included trials with an informative precue (80% of trials in experiment 1 and 100% of trials in experiment 2). Before sorting the behavioral data by response precision, we regressed out the influence of target tilt on the reproduction error. After sorting the data by response times or precision, we then calculated the same contralateral-versus-ipsilateral contrast of prospective action preparation as described above, separately for the delay intervals followed by a “fast” (< median response time) or “slow” (> median response time) response, as well as for trials with a “precise” (< median error) or “imprecise” (> median error) response after the memory probe.

#### Source analysis

Source analysis was identical to the analysis described in (*26*). We placed a 1-cm^3^ grid inside the generic Montreal Neurological Institute (MNI) T1 template brain and used a boundary element volume conduction model that describes how grid points projected to the generic 10-10 electrode positions. Spectral power in the predefined 8- to 12- and 13- to 30-Hz bands was localized using a frequency-domain beamformer (*62*). For each grid point, we calculated the normalized difference in power between trials in which the item/response hand was contralateral versus ipsilateral to the voxel—in the same way as we had done at the sensor level. Before source analysis, data were rereferenced to a common average reference. We note that the attribution of our neural lateralization signatures to “visual” or “motor” was based on the information that was considered in our experimental contrasts (visual item location and prospective response hand) not where this information localized to in the brain.

#### Statistical evaluation

Statistical evaluation of the contralateral-versus-ipsilateral contrasts in the EEG data was performed using a cluster-based permutation approach (*63*), which is ideal for evaluating the reliability of neural patterns across time and frequency. This approach effectively circumvents the multiple comparisons problem, by evaluating clusters that are observed in the group-level data against a single permutation distribution of the largest clusters that are found after permutation of the participant-specific trial averages. We performed 10,000 permutations and used default cluster settings. Specifically, we grouped adjacent same-signed data points that were significant in a mass univariate *t* test (two-sided, α = 0.05), and we used the sum of all *t* values in a cluster as the cluster statistic. The *P* value—that can be obtained for each cluster in the nonpermuted data (*63*)—is calculated as the proportion of permutations whose largest cluster exceeds that of the obtained cluster in the nonpermuted data. When zero permutations yielded a larger cluster than the one observed (as was the case for several of our observed clusters), the Monte Carlo *P* value is smaller than 1/*N* permutations (in our case *P* < 0.0001). We performed these analyses both on the full time-frequency maps and on full time courses in the predefined frequency bands of interest.

To quantify the relation between action encoding and later memory-guided behavior, we also quantified the strength of the action-encoding signature in the identified 400- to 800-ms action-encoding window (this window was set before evaluating the outcomes of this analysis, based on the relevant window identified in the preceding analyses) and used paired-samples *t* tests to compare fast and slow trials, as well as precise and imprecise trials (see the “Relation with behavior” section). Topographical sensor- and source-level analyses served only to confirm the plausibility of the results and to provide a richer depiction (*64*) and were not subjected to further statistical testing.

## SUPPLEMENTARY MATERIALS

Supplementary material for this article is available at http://advances.sciencemag.org/cgi/content/full/7/13/eabe8212/DC1

## Appendix

View/request a protocol for this paper from *Bio-protocol*.
